# Gamma Band Oscillations Reflect Sensory and Affective Dimensions of Pain

**DOI:** 10.3389/fneur.2021.695187

**Published:** 2022-01-10

**Authors:** Yuanyuan Lyu, Francesca Zidda, Stefan T. Radev, Hongcai Liu, Xiaoli Guo, Shanbao Tong, Herta Flor, Jamila Andoh

**Affiliations:** ^1^Department of Cognitive and Clinical Neuroscience, Medical Faculty Mannheim, Central Institute of Mental Health, Heidelberg University, Mannheim, Germany; ^2^School of Biomedical Engineering, Shanghai Jiao Tong University, Shanghai, China; ^3^Department of Psychiatry and Psychotherapy, Medical Faculty Mannheim, Central Institute of Mental Health, University of Heidelberg, Mannheim, Germany

**Keywords:** emotional valence, pain, self-reported pain ratings, gamma band oscillations (GBOs), priming

## Abstract

Pain is a multidimensional process, which can be modulated by emotions; however, the mechanisms underlying this modulation are unknown. We used pictures with different emotional valence (negative, positive, and neutral) as primes and applied electrical painful stimuli as targets to healthy participants. We assessed pain intensity and unpleasantness ratings and recorded electroencephalograms (EEGs). We found that pain unpleasantness and not pain intensity ratings were modulated by emotion, with increased ratings for negative and decreased ratings for positive pictures. We also found two consecutive gamma band oscillations (GBOs) related to pain processing from time frequency analyses of the EEG signals. The early GBO had a cortical distribution contralateral to the painful stimulus and its amplitude was positively correlated with intensity and unpleasantness ratings, but not with prime valence. The late GBO had a centroparietal distribution and its amplitude was larger for negative compared to neutral and positive pictures. The emotional modulation effect (negative vs. positive) of the late GBO amplitude was positively correlated with pain unpleasantness. The early GBO might reflect the overall pain perception, possibly involving the thalamocortical circuit, while the late GBO might be related to the affective dimension of pain and top-down-related processes.

## Introduction

Pain is an unpleasant sensory and emotional experience associated with potential or actual tissue damage or described in such terms. From this definition, it emerges that pain contains both a sensory-discriminative and an affective-motivational dimension (1, 2). The sensory-discriminative dimension refers to the intensity quality of pain, whereas the affective-motivational dimension reflects the unpleasantness of a painful experience and the associated tendency to avoid it (3–5). Although pain intensity and unpleasantness ratings are known to be highly correlated, experimental manipulations using various modalities (visual, auditory, and olfactory) showed a differential modulation of the two dimensions. For instance, pleasant compared with unpleasant odors could decrease pain unpleasantness but had little effect on pain intensity (6, 7). Listening to pleasant music, however, reduced both pain intensity and unpleasantness (8). In all these studies, presentations of emotional material and painful stimulation occurred simultaneously. Additionally, these studies used a relatively long trial duration (>6 s), which might introduce cognitive confounds to the emotional modulation of pain such as attentional processes. Thus, a special experimental paradigm, such as prime-target presentation, might be useful to reduce those attentional or cognitive factors on emotional modulation of pain.

Cortical oscillations, which can be extracted by frequency domain analysis from scalp electroencephalogram (EEG) signal, reflect synchronization of neuronal ensembles (9). Recently, a focus was put on the cortical oscillations related to pain (10), such as the lower bands, like alpha (8–13 Hz), beta (14–30 Hz), and also higher gamma band oscillations (GBOs) (30–100 Hz). For instance, the amplitude of GBO has been shown to be closely coupled with the perceived pain intensity, rather than the actual stimulus intensity (11–14), suggesting that GBO could reflect the sensory-discriminative dimension of pain. However, it remains controversial whether GBO also carries information about the affective dimension of pain perception and thus changes in emotional valence could also affect GBO (15–19).

In this study, we investigated the influence of emotional valence on pain perception using both pain rating and cortical oscillatory measures. We presented pictures of various types of emotional valence (negative, neutral, and positive) as primes and then applied painful electrical stimuli to healthy participants. Changes in pain perception were assessed using pain intensity and unpleasantness ratings. Based on previous literature, we expected that emotional valence would modulate pain ratings such that negative pictures would increase pain perception compared with positive pictures (20). For cortical oscillations, we expected that the amplitude of GBO would be positively correlated with pain ratings and would be also modulated by emotional valence, especially for the negative one. Finally, we expected a positive correlation between normalized pain ratings (i.e., negative vs. neutral and negative vs. positive) and normalized GBO amplitude (i.e., negative vs. neutral and negative vs. positive).

## Materials and Methods

### Participants

A total of 21 healthy subjects (age: 23.5 ± 2.6 years, 11 females) participated in this study. Participants were all right-handed (mean score of the sample = +95.6), as assessed using the Edinburgh Handedness Inventory (21) and had no history of mental or neurological disorders. The participants were informed about the purpose and the methods used in this study and gave signed informed consent. This study was approved by the Ethics Committee of the Medical Faculty Mannheim of Heidelberg University.

### Experimental Procedure

**Figure 1:**
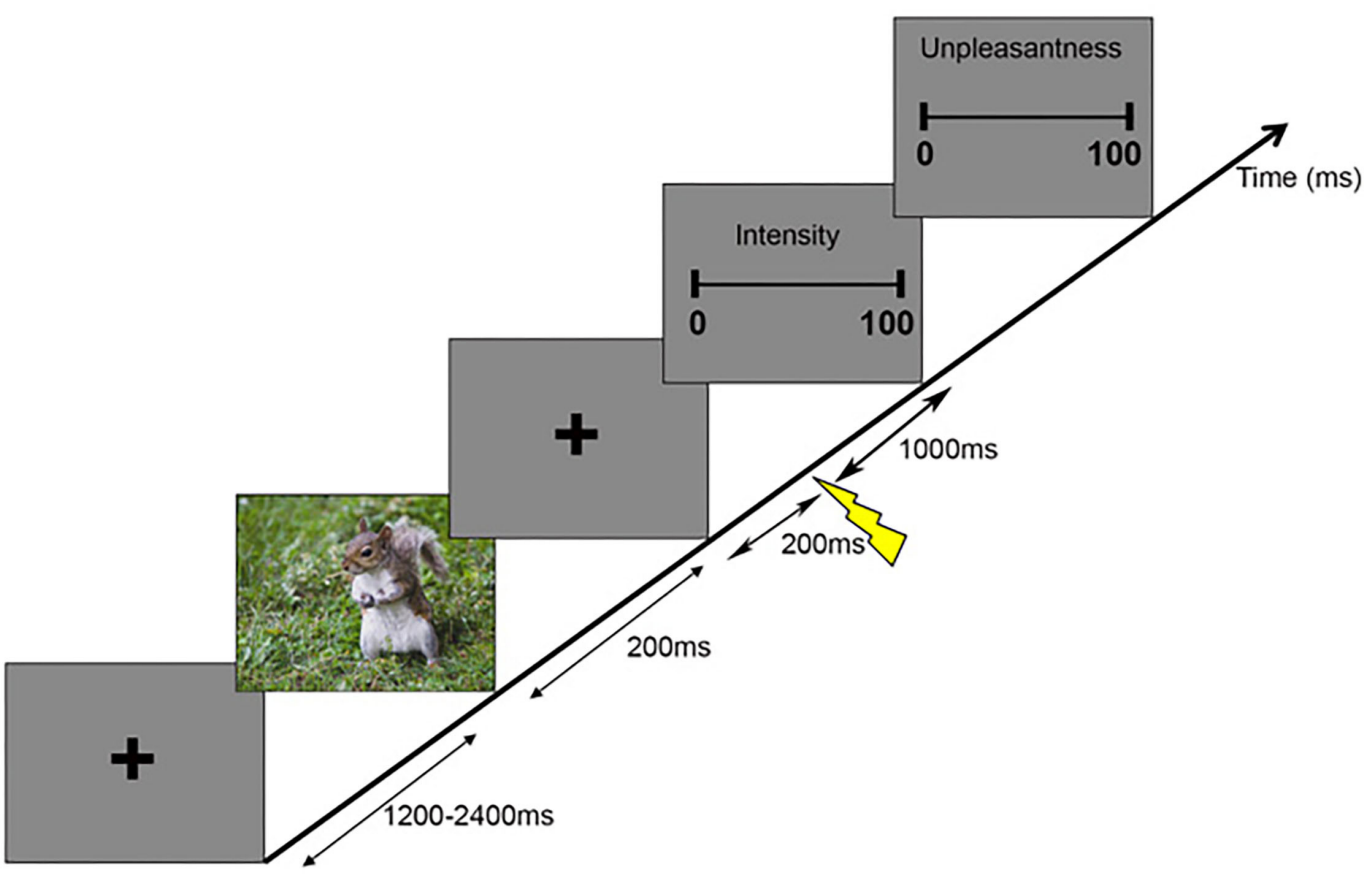
Schematic representation of the experimental paradigm. Each trial began with a fixation cross with a variable duration between 1,200 and 2,400 ms, followed by a picture lasting 200 ms. Then, another fixation cross was presented for 1,200 ms, during which painful stimuli were applied at a frequency of 3–7 Hz starting 200 ms after the prime picture. Then, two consecutive scales appeared, where participants indicated the intensity and unpleasantness of the perceived painful stimuli.

The participants sat in a comfortable chair in front of a monitor and the distance between the eyes and the monitor was ~50 cm. Before each trial, a fixation cross was presented in the center of a gray background for a randomized duration between 1,200 and 2,400 ms denoting the intertrial interval (Figure 1). Following the first fixation cross, a prime picture was displayed for 200 ms and was then replaced by a second fixation cross. After 200 ms, a painful electrical stimulus was applied at the left forearm by a bar electrode. After 1,000 ms from the onset of electrical stimulation, the participants were asked to perform ratings on the two consecutive visual analog scales (VASs): the first VAS related to the intensity of the pain (i.e., how intense was the painful stimulus?) ranging from no pain to most intense pain imaginable and the second VAS was used to rate the unpleasantness of pain (i.e., how unpleasant was the stimulus?) and ranged from not at all unpleasant to most unpleasant pain imaginable. The participants were asked to rate pain intensity and pain unpleasantness with the mean of a keyboard. They pressed the left and right arrow keys to adjust their ratings and then pressed the space bar to confirm. The prime pictures contained emotions of different valence (negative, neutral, or positive) and were taken from the International Affective Picture System (22)^1^. The pictures were selected based on normative ratings on the dimensions of affective valence (negative: 2.17 ± 0.36, neutral: 5.22 ± 0.55, and positive: 7.40 ± 0.40) and arousal (negative: 5.74 ± 0.51, neutral: 4.27 ± 0.59, and positive: 4.83 ± 0.73) and the rating scale ranged from 1 to 9, with 1 representing low pleasure and low arousal and 9 representing high pleasure and high arousal (23). We converted the rating scales to 0–100 for analysis. Although the arousal ratings of valence were different, we analyzed the results for a subset of stimuli with comparable arousal to show that arousal is not the main contributor to the present results (see the discussion). The three valence conditions were randomly presented over trials for each participant and consisted of 40 pictures each and each picture was only presented one time, i.e., 120 trials in total (40 × 3).

The electrical stimuli were generated by a constant stimulator (Digitimer® DS7A, Hertfordshire, UK). The Digitimer sends a square wave of 1 ms duration to a bar electrode attached to the right forearm of a participant. The intensity of the stimulus is determined by the voltage and duration of the square waves. For each participant, we measured the intensity of stimulus corresponding to the perception threshold, pain threshold, and pain tolerance three times before the experiment, respectively. For example, we increased the stimulation intensity until the participant perceived pain to determine the pain threshold. To make the electrical stimulus painful but tolerable, the chosen intensity was defined as mean pain threshold plus 80% of the difference between mean pain tolerance and mean pain threshold. To make sure the calculated intensity was robust and elicited reliable sensations before the experiment started, we tested the calculated intensity by asking participants to rate how painful was the stimuli perceived on the VAS, analogous to the one used for the pain intensity ratings. When the stimuli were not perceived as painful (i.e., with ratings inferior to 7/10), the intensity was increased until participants rated the stimuli with 7 or 8/10 on the VAS.

### EEG Acquisition and Analysis

The EEG signals were amplified by the BrainAmp amplifiers (BrainProducts GmbH, Munich, Germany, UK) and collected with BrainVision Recorder software, sampled at 1,000 Hz, and filtered online between 0.016 and 250 Hz. EEG was recorded using a 64-channel actiCAP with active Ag/silver chloride (AgCl) electrodes. Electrode positions on the cap were following the standard 10–10 system. Two more electrodes were used to record vertical and horizontal electro-oculograms to detect eye movements and blinks. The ground electrode was placed at AFz and the reference electrode was placed at FCz. Electrode impedance was kept at <20 kΩ, as suggested by the manufacturer. The active electrodes used here were demonstrated to be insensitive to moderate levels of impedance (<50 kΩ) when compared to passive electrodes for measurements such as EEG spectra (24).

Electroencephalogram data were preprocessed using EEGLAB version 15.3.6 (25). Data were first filtered using a 1-Hz high-pass filter and then interpolated the bad channels (percentage: 2.71 ± 2.06%). The filtered data were re-referenced to an average reference except for the eye electrodes and segmented in epochs from 1 s before to 2 s after the onset of the prime picture. Epochs with motion artifacts (i.e., 26.19 ± 17.19 epochs out of 120 epochs, i.e., 21.83 ± 14.33%) were rejected by visual inspection and the behavioral data of the rejected epochs were also excluded. After motion artifact rejection, there were 30.71 ± 6.05 negative epochs, 30.90 ± 6.67 neutral trials, and 32.14 ± 5.75 positive epochs per subject. The numbers of epochs showed no significant difference along with valence [*F*_(2, 40)_ = 1.707, *p* = 0.194]. Independent component analysis was applied to the clean epoched data and components representing artifactual non-brain activity were rejected, i.e., eye movements, cardiac activity, powerline noise (50 Hz), and electrical stimulation artifacts. Then, the preprocessed epochs were assigned to the three conditions based on the picture valence (negative, neutral, and positive).

Event-related spectral perturbation (ERSP) analyses (26) were performed using the newtimef() function in EEGLAB. Morlet wavelets transformation was applied to each single EEG epoch with a sliding window. The window had a length of 1,115 points (1,115 ms) and was shifted in a step of 1 data point (1 ms). The frequency range was from 3 to 100 Hz with a resolution of 1 Hz. The cycles of wavelets increased linearly from 3 cycles at the lowest frequency (3 Hz) to 20 cycles at the highest (100 Hz) to achieve a good trade-off between the time and frequency resolutions (27). The time-frequency transformed data were averaged across trials for each condition and each subject. The ERSP amplitude was calculated as 10 × log10 transformed multiples of amplitude change with respect to the baseline. The baseline was defined for each trial before averaging across trials, as the 442 time points before the prime pictures. Global grand averaged ERSPs were obtained by averaging ERSPs across all the prime pictures and all the participants. After visual inspection, we found two prominent GBOs with increased amplitude after the painful electrical stimulus in the stimulation in the following time-frequency windows and regions, i.e., (1) the early GBO, 420–500 ms, 35–70 Hz, right centroparietal area (FCz, FC2, FC4, Cz, C2, C4, CPz, CP2, CP4, Pz, P2, and P4) and (2) the late GBO, 500–660 ms, 60–95 Hz, middle centroparietal area (C3, C1, Cz, C2, C4, CP3, CP1, CPz, CP2, CP4, P3, P1, Pz, P2, and P4). For further analysis, the amplitude of each GBO was calculated by averaging the ERSP amplitudes across the above window and region for each participant and each prime valence.

We then determined the total GBO, defined by both phase-locked and non-phase-locked components. Meanwhile, the intertrial coherence (ITC) (25), also known as an event-related phase-locking value, was calculated for each GBO.

We also assessed the induced GBO defined as the non-phase-locked component of GBOs. For this purpose, we removed the ERSP signal from the EEG segments and calculated the induced ERSP using the same parameters as the one we used for the total ERSP. Then, we extracted the early- and late-induced GBO from the same time-frequency channel window for later statistical analysis.

### Statistical Analysis

Pain ratings and GBOs were inspected for normality using the Shapiro–Wilk test (see Supplementary Table S1).

Half of the variables were normally distributed (Shapiro–Wilk test, *p* > 0.05). Additionally, measures of skewness and kurtosis were used to evaluate deviation from normality. Absolute skewness values for all the variables were < 2, which is considered acceptable in order to prove normal distribution (28–31).

We also recalculated statistics using non-parametric test equivalents (software R package version 1.3.1093) (see Supplementary Table S5 ).

The pain ratings (intensity (INT) and unpleasantness (UNP)) and ERSP values in different time-frequency windows were analyzed using the one-way repeated measures ANOVA with prime valence (negative, neutral, and positive) as a within-subject factor. The one-way repeated measures ANOVA are considered fairly robust to deviations from normality as long as the levels of the within-subjects factor are similarly skewed. We, therefore, first used Mauchly's test of sphericity to test the assumption of sphericity and then used the Greenhouse–Geisser correction for the results when the sphericity assumption was not met. *Post-hoc* tests were corrected for multiple comparisons using the Bonferroni corrections.

To test whether the early and late GBOs shared the same characteristics of phase-locking activity, ITC values were analyzed using a 2 × 3 repeated measures ANOVA, taking prime valence (negative, neutral, and positive), and GBO (early and late) as within-subject factors. As the normative ratings of arousal differed between the negative and positive pictures, we selected for this study [*F*_(2, 117)_ = 58.04, *p* < 0.001] and we introduced arousal as a covariate in all the ANOVAs.

We also examined the relationship between the pain intensity and unpleasantness ratings and ERSPs using Spearman's rank correlation coefficient.

We also assessed the effects of habituation on the prime category as follows: for each prime valence, we divided the total number of trials (*n* = 40) by 4, resulting in 4 time points (10 trials per time point). We then carried out 3 × 4 repeated measured ANOVAs with time points and valence as within-subject factors on pain intensity and pain unpleasantness.

To quantify the emotional modulation effect, we carried out a normalization procedure on the pain ratings and GBO amplitude as follows:

Pain ratings (i.e., INT and UNP) were normalized by dividing them between negative and neutral prime valence [INT (neg/neu) and UNP (neg/neu)] and between negative and positive prime valence [(INT (neg/pos) and UNP (neg/pos)]. Then, we carried out correlation analyses between the normalized pain ratings and the normalized GBO amplitudes.

For GBO, since their amplitude was in the log domain, normalization was performed by subtracting GBO amplitude between the neutral and the negative prime valence [GBO (neg-neu)] and between the positive and the negative prime valence [GBO (neg-pos)]. Then, we carried out correlation analyses between the normalized pain ratings and the normalized GBO amplitudes.

Outliers were detected using the interquartile range (IQR), defined as the upper quartile minus the lower quartile. Values outside the range of the lower quartile-−1.5 × IQR to the upper quartile + 1.5 × IQR were excluded from all the analyses. The significance level was set at *p* < 0.05. All the data are presented as means ± SD.

## Results

### Pain Intensity and Unpleasantness Ratings

Pain intensity ratings were comparable across valence conditions [*F*_(2, 40)_ = 0.843, *p* = 0.371, negative: 31.49 ± 18.31, neutral: 31.70 ± 16.28, and positive: 30.41 ± 17.39]. In contrast, there was a main effect of prime valence on pain unpleasantness ratings [*F*_(2, 40)_ = 9.579, *p* = 0.006].

**Figure 2:**
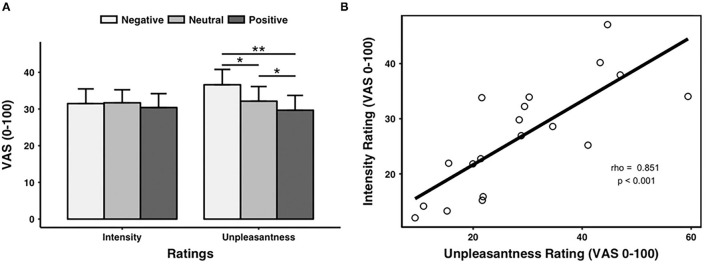
Pain ratings. **(A)** Ratings of pain intensity and unpleasantness for each prime valence (negative, neutral, and positive). The unpleasantness ratings showed a significant main effect of prime valence, while the intensity ratings did not show a significant main effect of prime valence. **(B)** Across all the pictures, the averaged intensity ratings were significantly positively correlated with the averaged unpleasantness ratings. VAS, visual analog scale. *p < 0.05, **p < 0.01. Error bars stand for SEs.

*Post-hoc* tests indicated that pain unpleasantness ratings were significantly higher for the negative (36.62 ± 19.11) than the neutral (32.15 ± 18.24, *p* = 0.001) and the positive (29.68 ± 18.52, *p* = 0.002) prime valence (for raw data, see Supplementary Table S2). In addition, pain unpleasantness ratings were significantly higher for the neutral than for the positive (*p* = 0.023) prime valence (as shown in Figure 2A, Supplementary Table S3 for mean pain ratings across prime valence).

We also found a positive correlation between the pain intensity and unpleasantness ratings (rho = 0.851, *p* < 0.001, *n* = 19, outliers: participants 3 and 8) (as shown in Figure 2B).

When excluding two outliers, the ANOVA results reported above led to similar results [i.e., *F*_(2, 36)_ = 1.83, *p* = 0.192 for pain intensity ratings and *F*_(2, 36)_ = 7.84, *p* < 0.001 for pain unpleasantness ratings].

For pain intensity ratings (INT), there was a trend toward significance for a main effect of time [*F*_(3, 60)_ = 2.734, *p* = 0.051], but no interaction between time and valence [*F*_(6, 120)_ = 0.564, *p* = 0.670]. For pain unpleasantness ratings (UNP), there was no main effect of time [*F*_(3, 60)_ = 2.182, *p* = 0.100] and no interaction between time and valence [*F*_(6, 120)_ = 0.925, *p* = 0.444] (refer to Supplementary Table S4 for the ratings calculated for each time point and for each valence).

### Total GBOs

**Figure 3:**
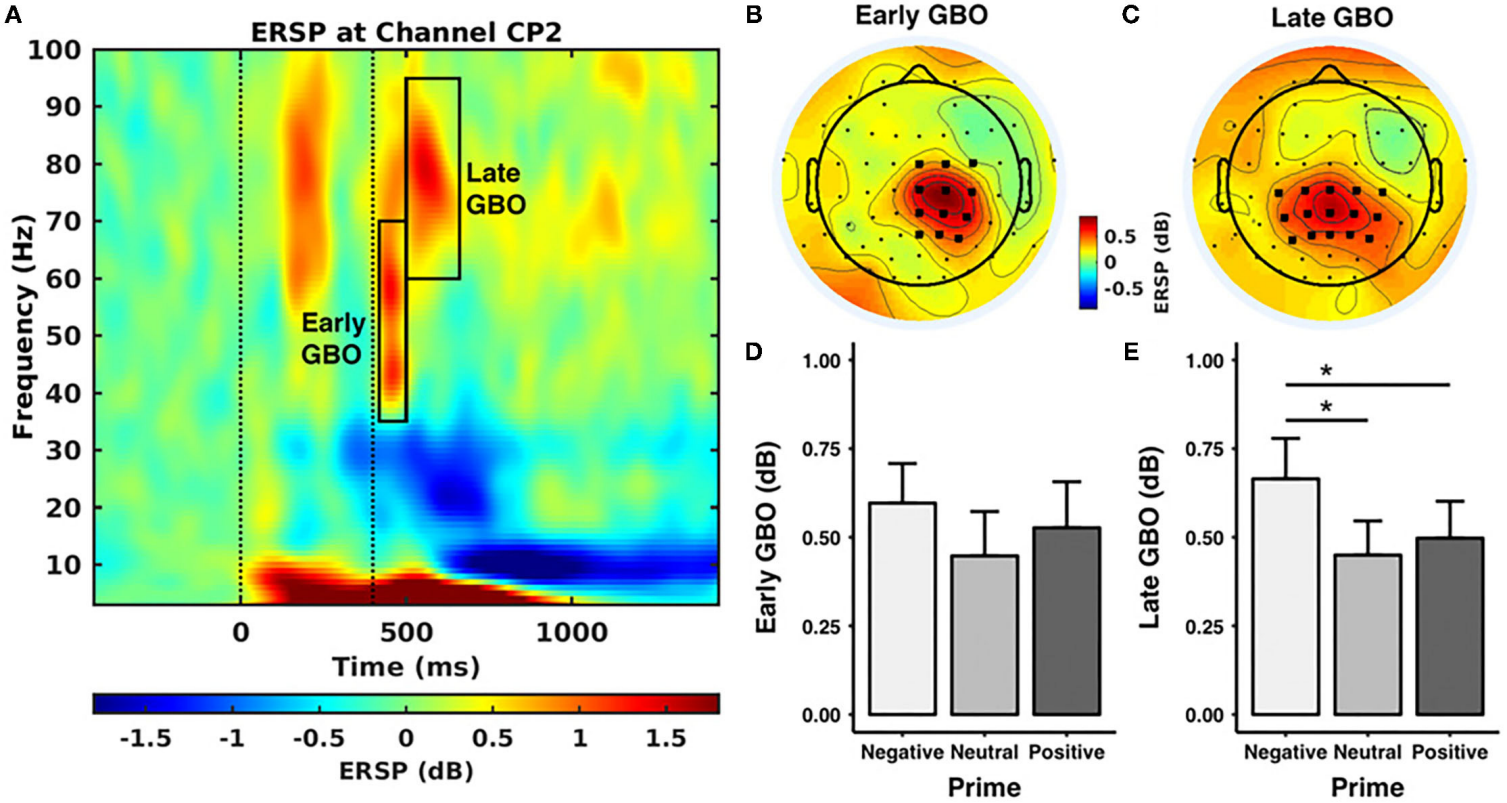
Gamma band oscillations (GBOs). **(A)** Event-related spectral perturbation (ERSP) at CP2 across all the pictures and all the subjects. The first dashed line stands for the onset of the prime stimulus and the second dashed line represents the onset of the electrical stimuli. The black rectangles indicate the time-frequency windows of the early and late GBOs. **(B,C)** The scalp distribution of the early and late GBOs. The early GBO had a central distribution contralateral to the stimulus location and the late GBO had a centroparietal distribution. The bold black dots indicate the regions of interest used in the statistical analyses. **(D,E)** The ERSP value of the GBO for each prime valence. The late GBO showed a significant main effect of prime valence, while the early GBO did not show a significant main effect of prime valence. **p < 0.05.

Figure 3A shows the event-related spectral perturbation as CP2 for all the subjects. After visual inspection, we found two prominent GBOs following the painful electrical stimuli. An early GBO (35–70 Hz) appeared in 20–100 ms after the electrical stimulus, centrally distributed in the hemisphere contralateral to the location of the stimulus application (Figure 3B). The late GBO, in a higher gamma band (60–95 Hz), appeared in 100–260 ms after the electrical stimuli, with a centroparietal distribution (Figure 3C).

The early GBO ~ 150–300 ms poststimulus mainly reflects the initial visual process of prime stimuli, which occurs before the pain stimuli and is, therefore, not related to emotion modulation of pain.

**Figure 4:**
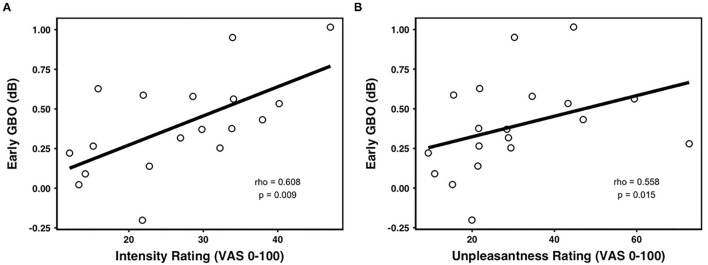
Correlations between the early GBOs and pain ratings. The mean early GBO was significantly positively correlated with **(A)** averaged intensity rating and **(B)** averaged unpleasantness rating across valence.

The amplitude of the early GBO was comparable across prime valences [*F*_(2, 40)_ = 2.099, *p* = 0.162, negative: 0.60 ± 0.51 dB, neutral: 0.45 ± 0.57 dB, positive: 0.53 ± 0.59 dB] (as shown in Figure 3D). In addition, the mean amplitude of the early GBO across valence conditions was positively correlated with the mean pain intensity rating across valence conditions (Figure 4A; rho = 0.608, *p* = 0.009, *n* = 18, outliers: participants 3, 6, and 8) and with the mean pain unpleasantness rating across valence conditions (Figure 4B; rho = 0.558, *p* = 0.015, *n* = 19, outliers: participants 3 and 6). Because the amplitude of the early GBO was not significantly different between the prime valences and, therefore, did not show any emotional modulation, we did not assess possible associations between the standardized amplitude of the early GBO and standardized measures of pain ratings, i.e., pain intensity (neg/neu or neg/pos).

The amplitude of the late GBO revealed a main effect of prime valence [*F*_(2, 40)_ = 6.151, *p* = 0.022; Figure 3E]. *Post-hoc* tests indicated that the amplitude of the late GBO for the negative prime valence (0.66 ± 0.52 dB) was larger than that for the neutral (0.45 ± 0.44 dB, *p* = 0.027) and positive (0.50 ± 0.48 dB, *p* = 0.046) prime valences. In addition, the amplitude of the late GBO was comparable between neutral and positive prime valences (*p* = 1.00). However, unlike the early GBO, the mean amplitude of the late GBO across valence conditions was not significantly correlated with the mean pain intensity ratings (rho = −0.018, *p* = 0.943, *n* = 19, outliers: participants 3 and 8) nor with the mean pain unpleasantness ratings (rho = 0.060, *p* = 0.797, *n* = 21, no outliers).

**Figure 5:**
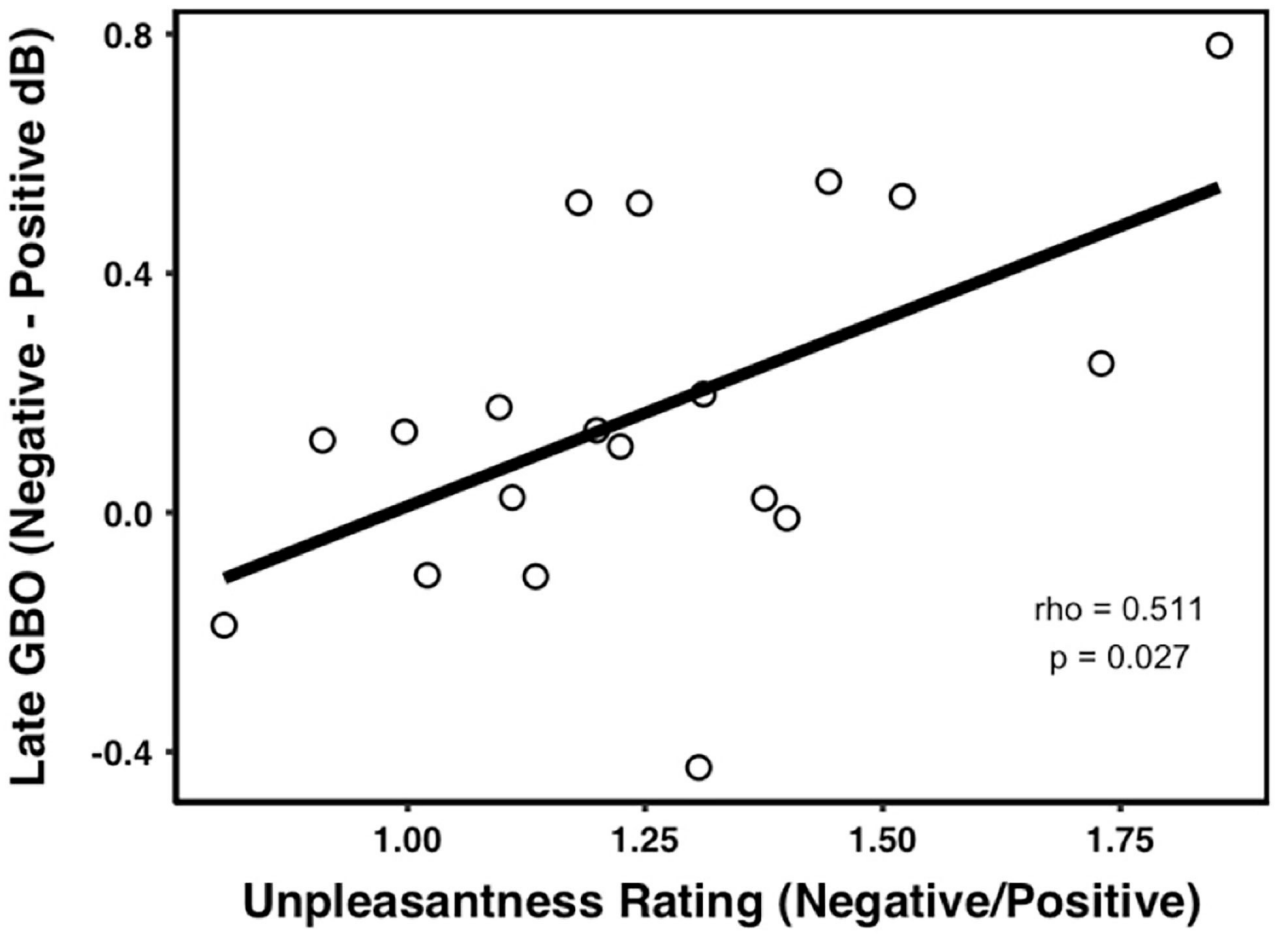
Correlations between the late GBOs and pain ratings. The amplitude of the late GBO was positively correlated with the unpleasantness rating in the negative prime condition compared with the positive prime.

The correlation analyses showed that there was no relationship between the normalized late GBO (neg-neu) amplitude and UNP (neg/neu) (rho = 0.270, *p* = 0.262, *n* = 19, outliers: participants 5 and 14). However, the normalized late GBO (neg-pos) amplitude was significantly positively correlated with UNP (neg/pos) (rho = 0.511, *p* = 0.027, *n* = 19, outliers: participants 5 and 12) (see Figure 5).

The non-parametric test results for pain ratings and the early and late GBOs followed the same trend as those reported using the one-way ANOVA (see Supplementary Table S5).

**Figure 6:**
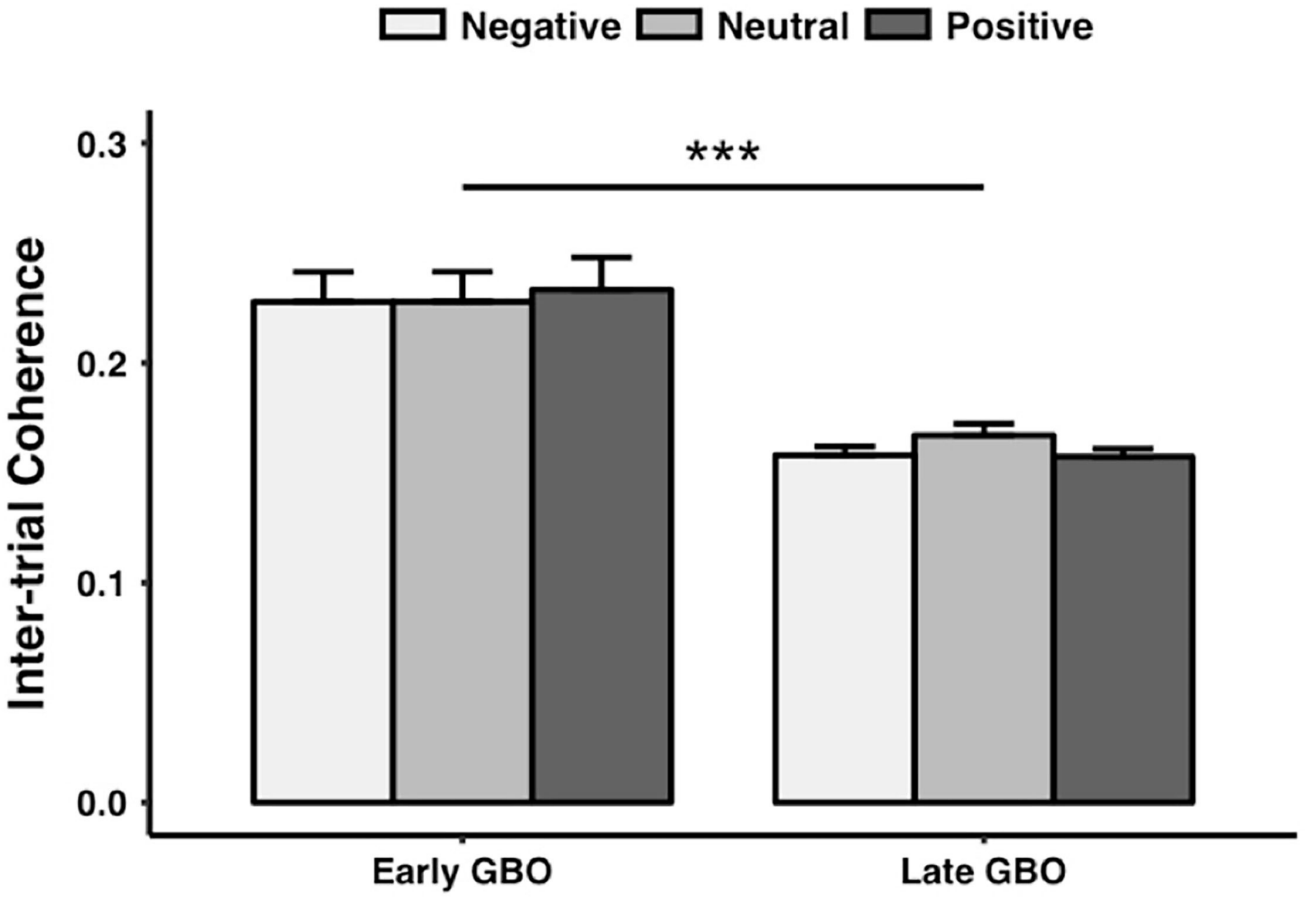
The intertrial coherence of the early GBO was significantly larger than that of the late GBO. ***p < 0.01.

Finally, ITC values exhibited a significant main effect of GBO [*F*_(2, 40)_ = 27.520, *p* < 0.001] but no significant main effect of prime valence [*F*_(2, 40)_ = 0.976, *p* = 0.384] and no interaction between GBO and prime valence [*F*_(2, 40)_ = 1.544, *p* = 0.226]. The early GBO (0.23 ± 0.06) was more phase locked than the late GBO (0.16 ± 0.02) (Figure 6).

### Induced GBOs

For the induced early GBO amplitude, the ANOVA showed an insignificant main effect of prime valence [*F*_(2, 40)_ = 1.374, *p* = 0.265]. Meanwhile, the induced early GBO amplitude was not significantly correlated with neither pain intensity rating (rho = 0.41, *p* = 0.63) nor pain unpleasantness rating (rho = 0.47, *p* = 0.19).

For the induced late GBO amplitude, the ANOVA showed a significant main effect of prime valence [*F*_(2, 40)_ = 6.547, *p* = 0.003]. *Post-hoc* analysis showed that the induced late GBO amplitude after negative prime (0.66 ± 0.52 dB) was significantly larger than the one after positive prime (0.44 ± 0.45 dB, *p* = 0.035) and neutral prime (0.50 ± 0.47 dB, *p* = 0.019). The normalized late GBO (neg-pos) amplitude was significantly positively correlated with UNP (neg/pos) (rho = 0.519, *p* = 0.024, *n* = 19, outliers: participants 5 and 12).

## Discussion

We investigated how the sensory and affective dimensions of pain were modulated by emotional valence using self-reports of pain and gamma band neural oscillations. Pain ratings showed that emotional valence affected pain unpleasantness, but not pain intensity.

Negative prime pictures increased pain unpleasantness, while positive prime pictures decreased pain unpleasantness. Although there was some habituation of the pain intensity ratings, they stayed in the painful range and did not significantly differ between the valence categories.

Moreover, we identified two consecutive GBOs following painful stimuli. The early GBO correlated with the overall pain intensity and pain unpleasantness ratings and was not influenced by emotional valence. On the other hand, the late GBO in the higher gamma band was modulated by emotional valence, particularly for the negative valence condition.

Only the pain unpleasantness ratings were significantly different across the three prime valences, indicating that the affective rather than the sensory dimension of pain was sensitive to the emotional pictures. The visual stimuli used in the current design were characterized by two dimensions: valence and arousal, but the modulation effect is most likely driven by the dimension of valence. First, in the subset analysis on positive and negative pictures with comparable arousal ratings, the negative pictures elicited significantly larger unpleasantness ratings than positive ones. Second, according to the distraction theory, the pictures with a high arousal rating (positive/negative) would trigger a decrease in pain perception than neural ones, which is not the case in our results (32–34). Our results add a new perspective to the current literature and the experimental design used in this study was intended to optimize the assessment of emotional modulation of pain. On the one hand, the painful stimuli used in this study were delivered without additional concomitant confounds. This may limit the interaction of additional cognitive factors such as attention as concomitant presentation of emotional stimuli with the delivery of painful stimuli was often used in previous studies (6, 17, 20, 35). On the other hand, the assessment of both intensity and unpleasantness pain ratings enabled us to differentiate between sensory and affective dimensions of pain. Indeed, some studies have not assessed both pain unpleasantness and intensity ratings (17, 36, 37) and might have merged the sensory and affective dimensions of pain.

Our findings are in line with the neuroimaging literature highlighting that pain is a multidimensional process, which led to the need to assess both its sensory and affective dimensions (1, 2, 38). Although selective modulation of pain intensity and unpleasantness by cognitive manipulation is widely recognized, the evidence underpinning this separability remains weak (39). In this study, Talbot et al. discuss possible biases in cognitive techniques and statistical methods that could underlie previously found a dissociation between sensory and affective dimensions of pain. Moreover, the authors suggest that cognitive processes might preferentially modulate the affective rather than the sensory dimension of pain. Current literature is still insufficient to provide arguments in favor or against this hypothesis. In this study, however, we used prime pictures to reduce cognitive processing such as attention during picture presentation and reported differential modulation of intensity and unpleasantness dimensions of pain by emotional primes. Further study is needed for manipulating various experimental factors, e.g., prime durations, order of pain ratings to better understand the mechanisms underlying differential regulation of pain intensity and unpleasantness.

With respect to the GBOs, we were able to show two consecutive GBOs following painful stimuli and several dissociations between them: the early GBO had a distribution widespread over the contralateral S1, while the late GBO was widespread over a large centroparietal area in the midline and appeared in a higher gamma band and at later time window. With respect to the behavioral measures, the early GBO encoded the overall perceived pain intensity and unpleasantness, while the late GBO was modulated by emotional valence. Moreover, the phase-locked value of the early GBO was significantly larger than the late GBO. When we only consider the induced GBO, the correlations between the early GBO and overall pain ratings did not reach a significant level, while modulatory effect and the relationship with unpleasantness rating still hold in the late GBO. Thus, the early and late GBO mainly originate from phase-locked component and non-phase-locked component, respectively, and might be mediated by different mechanisms.

The early GBO is most likely a time-frequency representation of the early complex N20-P30 wave of somatosensory evoked potential (SEP) elicited by the electrical stimulation of the upper limb. The N20-P30 is phase-locked and originates from the contralateral somatosensory cortex (40). A previous study showed that the median nerve SEP contained oscillation components ranging from 30 to 80 Hz (41). Our result showed that the phase-locked component of the early GBO encodes the perceived pain intensity and unpleasantness in the phasic experimental pain condition. Such findings are not surprising, since pain intensity and unpleasantness ratings were highly correlated. The early GBO may reflect the temporal binding of thalamocortical projections (1, 42). Simultaneous recordings from the ventral posterior medial nucleus of the thalamus and corresponding cortical columns showed that the thalamic GBO had a strong phase modulation to the cortical GBO evoked by brief single-whisker deflection in rats (43). Likewise, source analysis of magnetoencephalographic data in humans showed such coherent thalamocortical GBO in the auditory modality (44). Furthermore, our results showed that the early GBO was not significantly modulated by emotional valence.

Unlike previous reports indicating that GBO encodes the perceived pain intensity in a phasic pain condition (11–13, 16), our results showed that the late nonphase-locked GBO did not directly encode the pain perception, but was modulated by emotion valence. The direct comparison of the amplitude of the late GBO among the different prime valence revealed an increased response to negative than positive and neutral prime valence. The role of stimuli valence, especially negative items, has previously been shown to affect GBO in a passive viewing mode (45). Our results indicated that the negative valence from priming visual stimuli could also induce the higher GBO later in the pain perception process and may reflect a top-down modulation. Likewise, an EEG study presenting pain stimuli together with emotional facial expressions also showed an emotional modulation of GBO, in which the authors found facial expression fear elicited increased GBO compared with facial expression angry (17). Since synchrony in the gamma band is related to the communication between cortical areas (46), it can be speculated that the increased late GBO in the centroparietal area may represent upregulated descending pain processing pathway triggered by negative prime. Such a top-down modulation may also contribute to the increased pain unpleasantness rating. Moreover, the emotional modulation effect from negative to positive of the late GBO is significantly correlated to that of pain unpleasantness ratings. Negative affects facilitate avoidance-motivated behavior, while positive affects facilitate approach-motivated behavior (47). As an aversive stimulus, acute pain also triggers avoidance-motivated behavior (48). The late GBO might represent the avoidance-motivated behavior, as negative prime and pain would enhance the effect, while positive prime and pain would counteract the effect. Overall, the late GBO might reveal the emotional modulation in the affective dimension of pain perception.

Finally, our results are in agreement with a serial model of pain perception (49), as the early GBO seems to encode the overall pain intensity and unpleasantness, but the late GBO indicates the emotional modulation in the affective dimension occurs later. The early GBO would be fundamental to the late GBO. Further studies are needed to clarify the mechanisms underlying the GBOs in the emotional modulation of pain.

### Limitations

Some limitations need to be addressed in this study. The duration of the presentation of the pictures was relatively short (200 ms) compared with previous studies [2 s for (36) and 6 s for (20, 50)], because we intended to reduce cognitive processing such as attention during picture presentation. Our prime picture duration should, however, have been sufficient, since modulatory effects by emotions have been shown to last up to 700 ms in an event-related potential study (51), which is longer than our prime-target interval (400 ms).

We used a single intensity of stimulation, we therefore cannot preclude the possibility of dependence of priming effects on stimulus intensity (36). Our interpretation is, therefore, limited to a single stimulation intensity.

The electrical stimulation used in this study would inevitably activate the non-nociceptive system, while we targeted the nociceptive system. Our results showed the amplitudes of the early and late GBOs were associated with pain ratings, indicating the brain response following electrical stimulation carries nociceptive information. In future studies, it is better to use laser stimulation or intraepidermal electrical stimulation, which would selectively or largely preferentially activate cutaneous Aδ- and C-fiber nociceptors (52, 53). Alternatively, using non-painful electrical stimulation as a control condition could also work.

The GBO following electrical stimulation might be contaminated by the preceding visual-evoked brain activity. To decrease the potential effect, one could use visual pictures without electrical stimulation as a control condition (17).

Finally, our results showed that the overall early GBO amplitude was significantly correlated with the overall pain intensity and unpleasantness ratings across the emotional valences; thus, in order to dissociate pain intensity and unpleasantness ratings, we carried out partial correlations. The early GBO was not significantly correlated with the pain intensity rating (*p* = 0.58) when the unpleasantness rating was controlled for or the unpleasantness rating (*p* = 0.29) when the intensity rating was controlled for, showing, therefore, that the two pain dimensions strongly interact with each other and that both might contribute to the early GBO.

It is also important to note that the correlation analyses were carried out with outlier extraction. We used an outlier criterion (based on IQR) that resulted in obtaining different outliers for different analyses. This outcome highlights interindividual variability in experimental pain responses, possibly related to genetic and psychosocial factors [for study (54)].

## Conclusion

We showed that emotional valence modulated selectively the affective dimension of pain. Moreover, we observed that the early GBO might reflect the overall sensory discriminative and affective dimensions of pain, while the late GBO might reflect the emotional modulation in the affective dimension of pain. Pain perception seems to be composed of serial processes, defined by different temporal dynamics and spatial coding.

## Data Availability Statement

The raw data supporting the conclusions of this article will be made available by the authors, without undue reservation.

## Ethics Statement

The studies involving human participants were reviewed and approved by Ethics Committee of the Medical Faculty Mannheim of Heidelberg University. The patients/participants provided their written informed consent to participate in this study.

## Author Contributions

SR, FZ, and JA designed the experiments. SR, YL, and HL performed the experiments. YL, FZ, SR, and HL conducted the data analysis. YL and JA prepared figures and tables. YL, FZ, SR, HL, XG, ST, HF, and JA wrote the manuscript. All authors reviewed and approved the final version of the manuscript.

## Funding

This study was supported by a grant from EFIC Grünenthal (EGG) to JA, a grant from the Deutsche Forschungsgemeinschaft (SFB1158/B07) to HF and JA, and a fellowship by the China Scholarship Council (CSC) to YL and HL.

## Conflict of Interest

The authors declare that the research was conducted in the absence of any commercial or financial relationships that could be construed as a potential conflict of interest.

## Publisher's Note

All claims expressed in this article are solely those of the authors and do not necessarily represent those of their affiliated organizations, or those of the publisher, the editors and the reviewers. Any product that may be evaluated in this article, or claim that may be made by its manufacturer, is not guaranteed or endorsed by the publisher.

## Footnotes

^1^Picture numbers were: neutral (1,390, 1,903, 2,025, 2,032, 2,235, 2,372, 2,487, 2,514, 2,521, 5,900, 6,000, 7,011, 7,013, 7,018, 7,021, 7,033, 7,042, 7,044, 7,057, 7,058, 7,077, 7,081, 7,096, 7,137, 7,140, 7,183, 7,184, 7,188, 7,237, 7,248, 7,249, 7,513, 7,550, 7,560, 7,620, 7,632, 7,820, 7,830, 9,150, and 9,468); positive (1,410, 2,035, 2,045, 2,050, 2,057, 2,070, 2,150, 2,274, 2,311, 2,340, 2,352, 1,440, 2,360, 2,395, 2,550, 2,660, 4,640, 4,641, 5,220, 5,480, 5,825, 5,830, 1,463, 7,230, 7,260, 7,270, 7,330, 7,470, 8,120, 8,461, 8,496, 8,501, 8,502, 1,510, 8,540, 1,630, 1,710, 1,721, 1,750, and 1,999); and negative (2,301, 2,352, 2,710, 2,800, 2,900, 2,981, 3,016, 3,017, 3,051, 3,059, 3,061, 3,064, 3,168, 3,181, 3,185, 3,220, 3,225, 3,301, 3,550, 6,022, 6,213, 6,243, 6,415, 6,520, 6,560, 6,563, 9,040, 9,043, 9,075, 9,140, 9,181, 9,185, 9,253, 9,265, 9,332, 9,405, 9,571, 9,635, 9,800, and 9,902).
