# Supplementary material for: Gamma Band Oscillations Reflect Sensory and Affective Dimensions of Pain
Source: Front Neurol. 2022 Jan 10;12:695187. doi: 10.3389/fneur.2021.695187 (PMC8784749; doi:10.3389/fneur.2021.695187)
Supplement: Supplementary file 1 [file Table_1.docx]

**Supplementary material**

Yuanyuan Lyu, Francesca Zidda, Stefan Radev, Hongcai Liu, Xiaoli Guo, Shanbao Tong, Herta Flor, Jamila Andoh “Gamma Band Oscillations Reflect Sensory and Affective Dimensions of Pain”

***Table S1.***

*Shapiro-Wilk test, skewness and kurtosis for the variables pain ratings and GBOs*

|  | Shapiro-Wilk test  (p value) | Skewness | Kurtosis |
| --- | --- | --- | --- |
| Pain intensity ratings  Negative primes  Neutral primes  Positive primes | 0.002*  0.004*  0.009* | 1.45  1.41  1.27 | 4.72  4.80  4.03 |
| Pain unpleasantness ratings  Negative primes  Neutral primes  Positive primes | 0.032*  0.070  0.070 | 1.15  0.83  0.71 | 3.76  2.88  2.44 |
| Early GBOs  Negative primes  Neutral primes  Positive primes | 0.875  0.0002*  0.013* | 0.10  1.76  1.05 | 2.48  5.86  3.36 |
| Late GBOs  Negative primes  Neutral primes  Positive primes | 0.849  0.901  0.444 | 0.32  0.17  -0.43 | 2.32  2.26  2.41 |
