# Supplementary material for: Gamma Band Oscillations Reflect Sensory and Affective Dimensions of Pain
Source: Front Neurol. 2022 Jan 10;12:695187. doi: 10.3389/fneur.2021.695187 (PMC8784749; doi:10.3389/fneur.2021.695187)
Supplement: Supplementary file 2 [file Table_2.docx]

**Supplementary material**

Yuanyuan Lyu, Francesca Zidda, Stefan Radev, Hongcai Liu, Xiaoli Guo, Shanbao Tong, Herta Flor, Jamila Andoh “Gamma Band Oscillations Reflect Sensory and Affective Dimensions of Pain”

***Table S2***

*Mean (*± *SD) perception thresholds, pain thresholds and stimulation intensity used for n=11 participants. Please note that because of technical issues, the remaining data (i.e. pain tolerance) are not available. Each measurement was repeated three times.*

|  | Perception thr1 | Perception thr2 | Perception thr3 | Pain thr1 | Pain thr2 | Pain thr3 | Intensity used (mA) |
| --- | --- | --- | --- | --- | --- | --- | --- |
| Mean *(*± *SD)* | 15.0±6.5 | 14..4±7.4 | 14.1±6.9 | 49.4±18.1 | 55.8±22.0 | 60.2±31.5 | 84.1±36.3 |
| Range [min;max] | [5;24] | [3;24] | [2;22] | [20;70] | [22;88] | [27;128] | [20;130] |
