# Supplementary material for: Gamma Band Oscillations Reflect Sensory and Affective Dimensions of Pain
Source: Front Neurol. 2022 Jan 10;12:695187. doi: 10.3389/fneur.2021.695187 (PMC8784749; doi:10.3389/fneur.2021.695187)
Supplement: Supplementary file 3 [file Table_3.docx]

**Supplementary material**

Yuanyuan Lyu, Francesca Zidda, Stefan Radev, Hongcai Liu, Xiaoli Guo, Shanbao Tong, Herta Flor, Jamila Andoh “Gamma Band Oscillations Reflect Sensory and Affective Dimensions of Pain”

***Table S3***

*Mean (± SE) of the pain ratings (intensity and unpleasantness) for each valence category*

|  | Negative primes | Neutral primes | Positive primes |
| --- | --- | --- | --- |
| Pain intensity  ratings (±SE) | 31.49 ± 4.00 | 31.70 ± 3.55 | 30.41 ± 3.79 |
| Pain unpleasantness ratings (± SE) | 36.62 ± 4.17 | 32.15 ± 3.98 | 29.68 ± 4.04 |
