# Supplementary material for: Gamma Band Oscillations Reflect Sensory and Affective Dimensions of Pain
Source: Front Neurol. 2022 Jan 10;12:695187. doi: 10.3389/fneur.2021.695187 (PMC8784749; doi:10.3389/fneur.2021.695187)
Supplement: Supplementary file 4 [file Table_4.docx]

**Supplementary material**

Yuanyuan Lyu, Francesca Zidda, Stefan Radev, Hongcai Liu, Xiaoli Guo, Shanbao Tong, Herta Flor, Jamila Andoh “Gamma Band Oscillations Reflect Sensory and Affective Dimensions of Pain”

***Table S4***

*Mean (*± *SE) of pain intensity and pain unpleasantness ratings across time.*

| Pain intensity  ratings |  | Time 1 | Time 2 | Time 3 | Time 4 |
| --- | --- | --- | --- | --- | --- |
|  | Negative | 34.40±20.77 | 31.42±22.30 | 32.48±23.59 | 29.51±23.52 |
|  | Neutral | 33.53±20.17 | 32.61±21.39 | 30.93±21.66 | 29.60±22.40 |
|  | Positive | 33.59±21.16 | 30.73±22.18 | 29.27±22.34 | 28.17±21.62 |
| Pain unpleasantness ratings | Negative | 39.12±21.81 | 37.50±22.83 | 37.04±22.71 | 34.60±23.65 |
|  | Neutral | 33.03±21.18 | 32.24±22.69 | 30.35±22.54 | 30.94±23.02 |
|  | Positive | 32.17±22.98 | 30.35±23.53 | 27.72±21.66 | 27.30±21.91 |
