# Supplementary material for: Gamma Band Oscillations Reflect Sensory and Affective Dimensions of Pain
Source: Front Neurol. 2022 Jan 10;12:695187. doi: 10.3389/fneur.2021.695187 (PMC8784749; doi:10.3389/fneur.2021.695187)
Supplement: Supplementary file 5 [file Table_5.docx]

**Supplementary material**

Yuanyuan Lyu, Francesca Zidda, Stefan Radev, Hongcai Liu, Xiaoli Guo, Shanbao Tong, Herta Flor, Jamila Andoh “Gamma Band Oscillations Reflect Sensory and Affective Dimensions of Pain”

***Table S5***

Statistics using parametric (one-way repeated measures of variance, ANOVA) and the non-parametric test equivalents (Friedman test). In all cases, the non-parametric test results followed the same trend as those reported in the manuscript.

|  | One-way repeated measures of variance (ANOVA)  (p value) | Friedman Test  (p value) |
| --- | --- | --- |
| Pain intensity ratings  Negative vs neutral primes  Negative vs Positive primes  Positive vs Neutral primes | 0.83  0.29  0.13 | 0.51  0.13  0.13 |
| Pain unpleasantness ratings  Negative vs neutral primes  Negative vs Positive primes  Positive vs Neutral primes | 0.001*  0.002*  0.023* | 0.001*  0.001*  0.005* |
| Early GBOs  Negative vs neutral primes  Negative vs Positive primes  Positive vs Neutral primes | 0.16  0.41  0.37 | 0.13  0.83  0.83 |
| Late GBOs  Negative vs neutral primes  Negative vs Positive primes  Positive vs Neutral primes | 0.003*  0.046*  1.00 | 0.016*  0.050*  0.83 |
